# Economy of Labour in Hospital Management

**Published:** 1912-08-24

**Authors:** Alfred M. Hooper


### ECONOMY OF LABOUR IN HOSPITAL MANAGEMENT.

By ALFRED M. HOOPER.

In my article under the above heading, which appeared in The Hospital on July 13, I endeavoured to show how the use of printed forms, order books, etc., could be the means of saving labour in institutional administration. In the same issue appeared a paragraph entitled "The Value of Printed Forms," in which the question was raised as to possible objections to their use, the chief of which seemed to be the danger that the secretary's office might lose its individual character and become a mere registering bureau. I do not think much alarm need be felt of this happening. Anyone acquainted with the working of a hospital secretary's office will realise how impossible it would be to cope with its multifarious duties and inquiries solely by means of printed forms, inquiries that range from the explanation of the new Health Insurance Act to the recommendation of the best doctor, say, in the Cannibal Islands, for any particular disease. As to becoming a mere registering bureau, the more perfect the system of registration the better for the institution, for the saving in time and labour expended in attending to minor details should afford better opportunity to the officials for looking after the general administration and for conceiving and carrying out fresh ideas tending to improvement and the welfare of their institutions. The article was not, of course, meant to convey that every possible circumstance could be met by printed forms but to show how these could be of great assistance. On the other hand, I do not suppose that it was seriously suggested that forms could be entirely dispensed with, for to reduce such an idea to absurdity we should have to abolish temperature charts, history sheets, and the many other similar forms in daily use, to say nothing of leaving out the heading to our stationery. Can anyone imagine views of a business-like contributor to the hospital's funds on receiving acknowledgment other than on some recognised form of receipt?

### The Question of Printed Slips.

Mr. W. Fenwick's article on this subject, which appeared on July 27, shows how a special form of receipt book can be the means of providing a careful check on cash receipts, and I quite agree with him when he remarks "there are methods necessary and methods unnecessary"—indeed, I would go much further and maintain there are methods not only unnecessary but undesirable. I trust he will forgive a little friendly criticism if I venture to say that one of the methods he illustrates appears to me to come rather near the last category. I refer to the printed slips which he states are issued from the wards giving the condition, in various stages, of the patients who are designated by numbers. In the first place, I assume that these slips are issued at a stated time every day, therefore the condition recorded on them would remain the same for twenty-four hours. When we consider the changes that might occur in a very much shorter period, even if the slips were issued several times a day, it would seem that the information so obtained could not be very reliable. Again, porters and even nurses are only human and as such liable to error, and, though we cannot entirely eliminate this possibility, it is necessary to reduce the chance of mistakes to a minimum. A misplaced figure or misinterpretation of a number, given, say, through the telephone, may cause confusion leading to a reply at variance with the actual facts and thus be the means of causing unnecessary anxiety to the patients' relatives or friends.

A preferable method of answering inquiries is, I think, by that of the use of an "Emergency List" and "Daily Bulletin." On the former it should be the duty of the house medical officer to place the names of those patients who have undergone a serious operation recently, and until out of danger, and also the names of those who are seriously ill from other causes. On the latter to be recorded the condition of such patients; and *any serious change in it should be reported to the office at once* by the house officer. Inquiries as to patients other than those on the "Emergency List" are allowed to be answered by the sister of the ward or the night superintendent. Where a telephonic intercommunication system exists, connecting the wards and other departments of the hospital, these inquiries can quickly be answered. I may mention that it should be an instruction to the sister in charge of the ward as soon as the name of a patient has been entered on the "Emergency List" to communicate with the nearest relative or friend, who is then allowed to visit at the discretion of the medical attendant or sister.

### The Abuse of the Telephone.

While on the subject of inquiries, a point arises on which I have no doubt many hospital officials have found difficulty, that is how far the use of the telephone should be allowed. If no rule exists on the subject it will sometimes be found that so many inquiries are made by the various relatives and friends of some particular patient that the instrument is almost monopolised. Of course, in the cases of patients in actual danger every effort should be made to give information, but in ordinary circumstances it is as well to allow telephonic inquiries from only the nearest relatives, others being requested to call or write.

Another point I would like to refer to in Mr. Fenwick's article is the method he gives of classifying accounts, which he describes as being done by placing them under cards bearing the mainheads of accounts as arranged by the uniform system, but this involves resorting under the subheads. He makes also no mention of filing. I have found the following a satisfactory system. In the first place, file all accounts, invoices, etc., as soon as they are received under an alphabetical index, according to the name of the supplier of the goods, so that, if required, any person's account can be easily found, then when the time for payment arrives each firm's account, invoices, etc., will be found together. When these have been classified under their proper subheadings, transfer to another file, having instead of an alphabetical an index bearing the various sub-heads. You will thus have your accounts grouped together under their proper headings, and when the book entries are made this will be found a great saving of labour. In practice it will not be found necessary to have an index for every subhead, for where there are only one or two accounts to include they may be put under an index marked "Various."

A Final Suggestion.

There are many kinds of filing cabinets on the market, but I have found the arch method the most practical, for although each account requires to be perforated, the labour involved is recompensed by their position being secure, whereas with the loose system the papers become too easily disarranged.

With regard to my suggestion that a recognised set of forms universally adopted by hospitals would tend to perfection in management, I may add that it should also be an economy, since the purchase of the very large quantities that would be required should bring about considerable reduction in cost. I suppose that no hospital official will claim that his methods are incapable of improvement, and it is only by comparison with systems in use at other institutions that we can judge of the value of our own. It would, therefore, be interesting and instructive if hospital managers would give the benefit of their experience through the medium of the columns of this Journal.

